# Muscle fat infiltration and its relation with pain intensity, disability, and cervical curvature in individuals with nonspecific neck pain: A systematic review study

**DOI:** 10.1002/pmrj.70064

**Published:** 2026-01-12

**Authors:** Hardianty Andi Munawarah Abduh, Hironobu Kuruma, Takuya Otsuka, Aufa Miftah Firdausy

**Affiliations:** ^1^ Department of Physical Therapy, Faculty of Human Health Science Tokyo Metropolitan University Hachioji Japan; ^2^ Department of Physiotherapy, Health Polytechnic of Makassar Makassar Indonesia; ^3^ Physioactive Indonesia South Jakarta Indonesia

## Abstract

**Objective:**

To review and analyze the association between muscle fat infiltration (MFI) and pain intensity, disability, and cervical alignment in individuals with nonspecific neck pain.

**Type:**

Systematic review study.

**Literature Survey:**

Nonspecific neck pain is associated with muscular changes, including fatty infiltration, which may contribute to chronic symptoms and functional limitations. Prior studies have examined morphological muscle changes across various neck pain populations; however, specific evidence regarding the relationship between MFI and clinical outcomes in nonspecific neck pain remains limited and inconsistent.

**Methodology:**

A systematic search of the Web of Science, PubMed, MEDLINE, and CINAHL databases was performed. This analysis included analytical cross‐sectional studies published from 2003 to May 2025 that used structural medical imaging to examine fatty infiltration of neck muscles in participants aged 18‐65 years with nonspecific neck pain lasting >3 months. Only articles written in English that were accessible to the authors were considered. Studies were excluded if they did not meet any of the aforementioned criteria.

**Synthesis:**

Across included studies (n=4), higher MFI in cervical extensor muscles was consistently associated with greater pain intensity and disability. Evidence for a relationship between MFI and cervical alignment was inconsistent, with some studies reporting a negative association between MFI and cervical lordosis, while others found no correlation. Variability in imaging methods and clinical measures contributed to heterogeneity between studies.

**Conclusion:**

The study results suggest a correlation between fatty infiltration and nonspecific neck pain features, such as pain level and neck disability, whereas cervical lordosis does not appear to be correlated. These findings highlight the importance of rehabilitation programs to reduce MFI and enhance muscle functions, which may alleviate symptoms.

## INTRODUCTION

Neck pain is a significant public health issue affecting a considerable proportion of the general population.^1^ Psychological factors including long‐term stress, lack of social support, anxiety, and depression, are significant risk factors for neck pain.^2^ Neck pain encompasses nonspecific neck pain (NSNP) and pain‐associated disorders.

NSNP is cervical pain without an apparent underlying pathological etiology. This condition causes restricted neck movement, muscle dysfunction, myofascial pain syndrome, and work stress, which negatively affect health‐related quality of life.^3^ It is reported that a significant proportion of individuals with chronic idiopathic neck pain (CINP) exhibit persistent symptoms or experience a recurrence of pain within a 5‐year time frame.^4^ Additionally, epidemiological studies show a high prevalence of NSNP among adolescents, with lifetime prevalence reported at 51%, 12‐month prevalence at 39.8%, 6‐month prevalence at 32.3%, and point prevalence at 5.6%.^5^ Several factors, including age, sedentary behavior, poor posture, and physical inactivity, contribute to an increased risk of this condition.^5^, ^6^ To effectively address the high prevalence of NSNP, it is essential to implement preventive measures, such as postural education, limiting screen time, and promoting physical activity.

Muscle fat infiltration (MFI), also known as intermuscular adipose tissue, is a pathological condition characterized by the accumulation of excess fat within skeletal muscle. This condition is commonly associated with the aging process and has been identified as a key contributor to muscle atrophy and reduced muscle mass.^7^, ^8^, ^9^ Intermuscular adipose tissue is associated with high levels of proinflammatory cytokines within the fatty infiltration, which can lead to poor function and a decrease in muscle strength. This, in turn, may have implications for overall physical health and mobility.^10^, ^11^, ^12^, ^13^

MFI in paraspinal muscles, particularly the erector spinae and multifidus, has emerged as an important radiological and clinical feature in spinal disorders. Studies have shown that fatty degeneration in these muscles is associated with reduced spinal stability and poorer clinical outcomes in conditions such as low back pain and lumbar spondylolisthesis.^14^, ^15^ In lumbar pathologies, the degree of MFI in the erector spinae has been proposed as a potential biomarker for predicting surgical need, with each incremental increase associated with a higher likelihood of surgery.^15^ Similarly, research comparing symptomatic and asymptomatic individuals revealed that patients with low back pain exhibit significantly more MFI in the upper lumbar erector spinae compared to controls.^14^

Moreover, a reciprocal relationship has been observed between the multifidus and the psoas at the L4–L5 level. In women with low back pain, greater MFI in the multifidus was associated with compensatory sparing of the psoas, suggesting an adaptive stabilizing mechanism of deep trunk muscles to maintain segmental control.^16^ These findings underscore the broader role of paraspinal muscle quality in spinal health and justify closer investigation into how MFI may affect function and clinical outcomes across spinal regions.

A previous study found that higher levels of MFI in extensor and flexor neck muscles were associated with a higher level of disability in people with degenerative cervical myelopathy.^17^ Moreover, there is a study that examined the relationship between MFI and clinical status in patients with spondylotic myelopathy.^18^ This study found that patients with higher levels of MFI in the multifidus muscle tended to have more disability, as measured by the Nurick score. However, the study did not find a correlation between MFI and disability scores measured by the neck disability index. Furthermore, in adult patients with spinal deformities, researchers found a relationship between global sagittal malalignment and MFI of the posterior vertebral muscles in the lumbar and thoracic spine, as detected by computed tomography (CT) scans.^19^ The study suggests that fatty degeneration could weaken the spine stabilizers, impairing the patient's ability to compensate and exacerbating the malalignment while increasing muscle stress and advancing the degenerative process. Nonetheless, studies investigating the link between cervical malalignment and chronic neck pain yielded controversial findings.

Magnetic resonance imaging (MRI) is considered the most reliable method for evaluating spinal muscles due to its high resolution, ability to provide soft tissue contrast, and superior visualization of landmarks.^20^ According to the Gautallier classification, semiquantitative methods were used in evaluating intramuscular fat infiltration.^21^, ^22^ Nevertheless, this may fail to identify small amounts of intramuscular fat infiltration due to subjective visual observation.^23^ Alternatively, CT scan can also be used for evaluating MFI as it is a viable option with good reliability for evaluating the cross‐sectional area and density of paraspinal muscles with a shorter examination time.^24^, ^25^ Both CT and MRI were equally effective in evaluating the atrophy of the multifidus and assessing the MFI in chronic low back pain.^24^ Furthermore, ultrasound is also an effective tool for detecting fat accumulation, especially in rotator cuff muscles. It has advantages over MRI such as immediate evaluation, insensitivity to metal implants, and cost‐effectiveness. However, interpreting ultrasound results requires expertise, and performing it on obese patients can be challenging due to limited wave penetration.^26^ Therefore, the selection of a suitable method is contingent upon the precise muscle region under consideration for examination.

A recent study using systematic review and meta‐analysis has discovered a close relationship between MFI in the thigh muscles of individuals with knee osteoarthritis and systemic inflammation, metabolic impairments, poorer physical performance, muscle impairment, and muscle dysfunction.^27^ Similarly, another systematic review study delved into the changes in morphology, including MFI, in patients with chronic neck pain. The study revealed some evidence for increased fat in the flexors and extensors of individuals with whiplash‐associated disorder (WAD) and CINP.^28^ However, previous reviews have primarily investigated general muscle morphology changes and have included both WAD and NSNP populations.^28^, ^29^ The extent to which MFI correlates with clinical features such as pain intensity, functional impairment, and cervical alignment in NSNP remains unclear. MFI may contribute to higher pain and disability levels by impairing neuromuscular function, increasing muscle stiffness, and promoting local inflammation,^30^, ^31^, ^32^ all of which could disrupt cervical alignment and movement patterns. Understanding these mechanisms will help clarify the role of MFI in NSNP and its potential impact on rehabilitation strategies. Our systematic review aims to address this gap by specifically focusing on the relationship between MFI and these clinical outcomes, including pain intensity, disability, and cervical alignment in individuals with NSNP.

## METHODS

**FIGURE 1:**
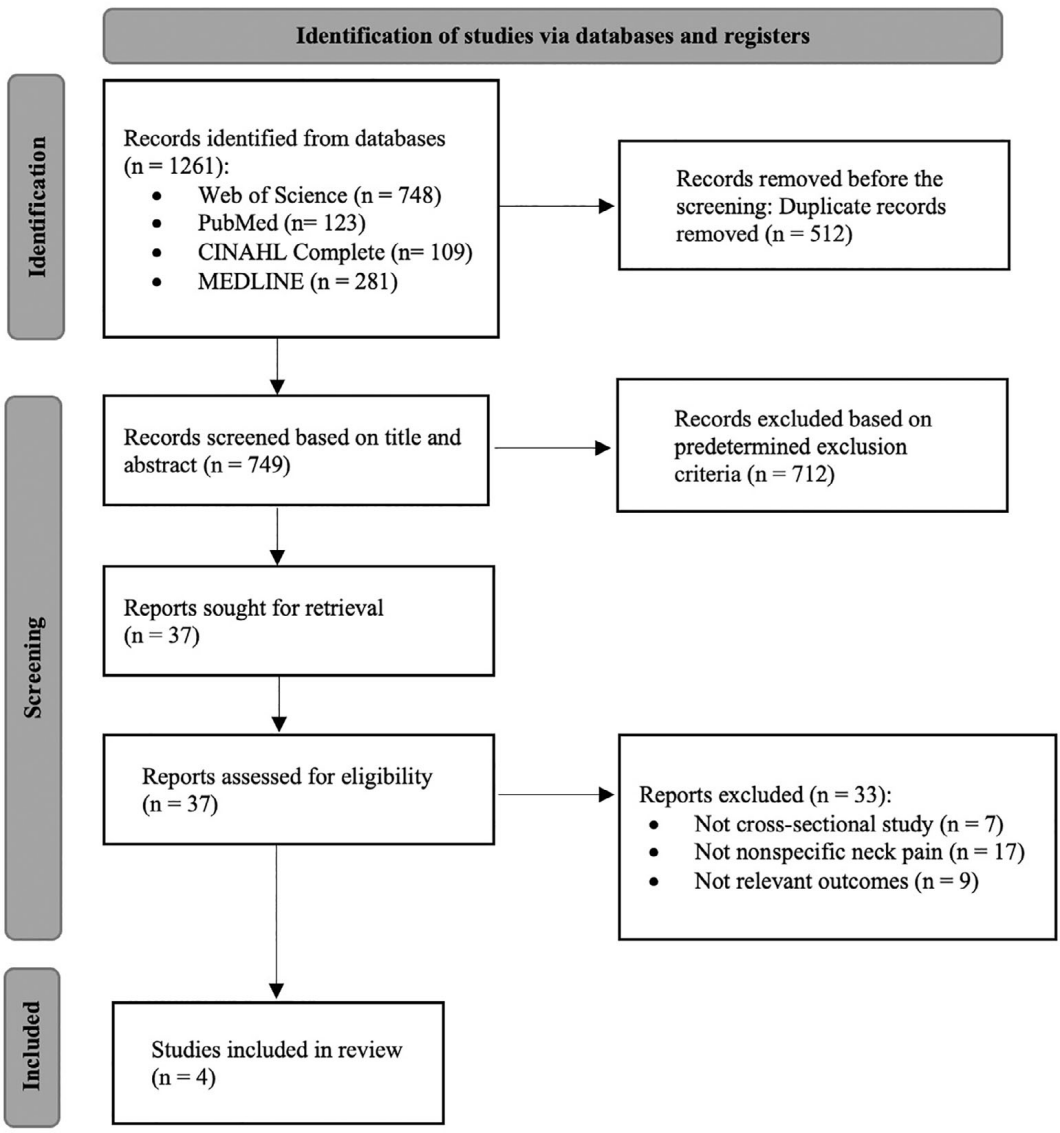
Preferred Reporting Items for Systematic Reviews and Meta‐Analyses (PRISMA) flow diagram showing the selection process for studies included in the systematic review.

This systematic review was conducted in strict compliance with the Preferred Reporting Items for Systematic Reviews and Meta‐Analyses (PRISMA) guidelines,^33^ considered the gold standard for conducting systematic reviews. The search results are presented in Figure 1.

The systematic review protocol specifying inclusion criteria and analysis methods was registered in advance on the International Prospective Register of Systematic Reviews (registration number: CRD42023454322).

### Criteria for eligibility, sources of information, and search strategy

Two reviewers (H.A.M.A. and T.O.) conducted a literature search in four electronic databases, Web of Science, PubMed, MEDLINE, and CINAHL, between January 1, 2003, and May 30, 2025.

The search strategy used MeSH (Medical Subject Headings) terms and free‐text words based on the participants, interventions, comparator, outcomes, study (PICOS) question. Study participants were required to have neck pain, chronic neck pain, or NSNP. The intervention included medical imaging techniques, such as MRI, CT, and ultrasound. The outcome was required to include differences between fat infiltration and pain intensity, disability, and cervical alignment, as defined by the MeSH terms: Neck Pain, Pain Measurement, Disability Evaluation, and Spinal Curvatures. The PICO‐based search strategy is summarized in Table 1, with the full database‐specific search strings available in the Appendix S1.

**TABLE 1:** Strategy to search keywords.

P (Patients)		I (Intervention)		O (Outcome)
Neck pain OR Chronic neck pain OR Nonspecific neck pain OR NSNP OR Chronic nonspecific neck pain OR CNSNP	AND	Magnetic resonance imaging OR Ultrasonography OR Computed tomography	AND	Muscle fat infiltration OR Fat infiltration OR Fat infiltration area OR FIA OR Neck muscles

*Note*: This study uses Boolean terms (AND and OR) to separate the distinct keywords.

The articles were chosen based on the following inclusion criteria: (1) studies including patients with NSNP or CINP, (2) studies that used structural medical imaging techniques (MRI, CT, and ultrasonography), (3) the measurement outcome of the studies consisting of fatty infiltration of the neck muscles, (4) an analytical cross‐sectional study, and (5) only English language studies. Studies were excluded if they reported participants with cervical injury or whiplash injury, detectable pathological spinal conditions, and systemic disorders (inflammatory disease, cancer, neurological disease, and immune deficiency) and did not meet all five inclusion criteria.

First, the articles were screened based on their titles and abstracts. Eligible articles were retrieved, and full texts were screened for the inclusion criteria.

### Data items and collection

An evidence table was created to summarize each article. The included articles were analyzed for author name, year of publication, patient characteristics, measurement technique, MFI measurement method, and primary results. Additionally, because no meta‐analysis was conducted, statistical tests for homogeneity were not applicable. Instead, heterogeneity was addressed descriptively by comparing variations in outcome measures (pain intensity, disability, and cervical alignment) and imaging methods used to quantify MFI.

### Risk of bias in individual studies

Two researchers (H.A.M.A. and A.M.F.) used the Joanna Briggs Institute critical appraisal checklist for analytical cross‐sectional studies.^34^ Each study was evaluated by two examiners using specific criteria to assess the level of bias. A high risk of bias was determined if the number of positive answers was ≤49%. A moderate risk of bias was identified when the level of bias was between 50% and 69%. Conversely, a low risk of bias was reported when the positive answers exceeded 70%.^35^

After reviewing the selected articles, the researchers compared their ratings and analyzed any discrepancies. In cases of disagreement, the reviewers reexamined the papers and discussed disparities until a consensus was reached.

## RESULTS

### Study selection

The search yielded 1261 references from four sources as follows: Web of Science (748), PubMed (123), CINAHL Complete (109), and MEDLINE (281). Four studies remained after excluding duplicates and conducting two screening phases based on the selection criteria. The complete information regarding the selection process is shown in Figure 1.

### Study characteristics

The study population ranged in age from 25 to 56 years. All included studies investigated females and males.^36^, ^37^, ^38^, ^39^ Two studies examined both flexor and extensor muscles,^36^, ^37^ whereas two others investigated only extensor muscles.^38^, ^39^ The average age of each patient group varied between 35.8 and 47.3 years, and the average body mass index varied between 24.6 and 26.9 kg/m^2^.

### Measurement of MFI

The assessment MFI in the four studies included primarily relied on MRI‐based techniques.^36^, ^37^, ^38^, ^39^ Two of the studies employed a semiquantitative approach with visual grading.^36^, ^37^ Specifically, one study followed the Goutallier classification using axial T1‐weighted imaging,^36^ and the other assessed fat signal intensity on T2‐weighted imaging.^37^

Additionally, two other studies used quantitative methods.^38^, ^39^ One of these used the fat‐to‐muscle signal intensity ratio on axial T2‐weighted imaging,^38^ and the other employed a threshold‐based segmentation technique to differentiate between fat and muscle tissue, providing objective measures of fatty infiltration.^39^ The heterogeneity in assessment methods is summarized in Table 2.

**TABLE 2:** Evidence table of the included studies.

Article	Population	Measurement technique	MFI measurement method	Main findings
Grondin, et al. 2022 ^36^	25 participants (5 males, 20 females; age: 47.3 ± 9.6 years; height: 1.66 ± 0.1 m; and BMI: 24.6 ± 5.2 kg/m^2^) CNSNP ≥3 months	MRI (Siemens, Erlangen, Germany)	Semiquantitative assessment using visual grading of fat replacement in muscle (Goutallier classification) on axial T1‐weighted MRI	*Neck pain level*: no outcomes *Neck disability level*: Fat infiltration in multifidus muscle was related to NDI, w longus colli muscle was not. *Cervical curvature*: no outcomes
Kim et al. 2017^38^	56 participants (28 males, 28 females; age: 39.7 ± 9.0 years; and BMI: 23.8 ± 1.8 kg/m^2^) Cervicalgia ≥3 months	MRI (General Electric, Milwaukee, WI, USA)	Quantitative assessment using fat‐to‐muscle signal intensity ratio using T2‐weighted axial MRI	*Neck pain level*: Fat infiltration in the extensor muscle was related to high VAS. *Neck disability level*: Fat infiltration in the extensor muscle was related to the NDI score. *Cervical curvature*: there was a negative correlation between cervical lordosis and fat infiltration in the cervical extensor muscles.
Huang et al. 2022 ^37^	55 participants (26 males, 29 females; age: 35.8 ± 8.7 years; and BMI: 26.9 ± 1.8 kg/m^2^) CNSNP ≥3 months	MRI (Siemens Avanto)	Semi‐quantitative assessment using visual grading of fat signal intensity in muscle on T2‐weighted MRI	*Neck pain level*: Fat infiltration in the right extensor muscles was related to NRS, whereas the left side was not. *Neck disability level*: Fat infiltration in the superficial extensor muscles and deep cervical paraspinal muscle, both flexor and extensor, was related to NPQ score. *Cervical curvature*: Fat infiltration in the deep extensor muscles and the cervical curvature were negatively correlated.
Snodgrass et al. 2022 ^39^	47 participants (25 males, 22 females; age: 36.80 ± 9.80 years; and BMI: 25.6 ± 4.2 kg/m^2^) CNSNP ≥3 months	MRI (Siemens Magnetom Prisma 3 Tesla Scanner)	Quantitative assessment using a threshold‐based segmentation technique on axial T1‐weighted MRI	*Neck pain level*: Fat infiltration in Multifidus, including MFSS was related to VAS. *Neck disability level*: no outcome. *Cervical curvature*: no outcome.

Abbreviations: BMI, body mass index; CNSNP, chronic nonspecific neck pain; MFI, muscle fat infiltration; MFSS, semispinalis cervicis; MRI, magnetic resonance imaging; NDI, neck disability index; NPQ, Northwick Park Questionnaire; NRS, numeric pain rating scale; VAS, visual analog scale.

### Extracted outcome data

Table 2 presents the outcome data obtained in each study.

### Risk of bias assessment

A detailed description of the risk of bias is presented in Table 3. Three studies were rated as low risk owing to the possibility of biased assessment, and one study was rated as high risk owing to limitations in design and implementation. In addition, all studies scored well on describing the criteria for inclusion in the patient population. However, two of the studies did not provide valid and reliable exposure measurements.

**TABLE 3:** Risk of bias assessment of cross‐sectional studies (JBI critical appraisal checklist for analytical cross‐sectional studies).

Author	Items on Joanna Briggs Institute checklist								Raw score and %	Risk
	Q1	Q2	Q3	Q4	Q5	Q6	Q7	Q8		
Grondin et al. 2022 ^36^	1	1	1	1	1	1	1	1	8/8 = 100%	Low
Kim et al. 2017 ^38^	1	1	N/A	N/A	0	N/A	N/A	U	2/8 = 25.0%	High
Huang et al. 2022 ^37^	1	1	1	1	1	1	1	1	8/8 = 100%	Low
Snodgrass et al. 2022 ^39^	1	U	N/A	1	1	1	1	1	6/8 = 75.0%	Low

*Note*: JBI criteria to be scored: Q1. Were the inclusion criteria clearly defined? Q2. Were the study participants and settings described in detail? Q3. Was the exposure measured in a valid and reliable manner? Q4. Were objective standard criteria used to measure this condition? Q5. Were the confounding factors identified? Q6. Were strategies used to deal with the confounding factors stated? Q7. Were the measured outcomes valid and reliable? Q8. Was considered appropriate for statistical analysis? 1 = Yes, 0 = No, U = Unclear, N/A = Not Applicable. Abbreviation: JBI, Joanna Briggs Institute.

### Synthesis of result

Four studies investigated the level of fatty infiltration in the cervical flexors and extensors.^36^, ^37^, ^38^, ^39^ Three studies investigated fatty infiltration in chronic NSNP,^36^, ^37^, ^38^ and another evaluated the quantity of fatty infiltration in CINP.^39^ Increased fat infiltration in the cervical extensors was significantly associated with higher visual analogue scale scores.^38^ It has also been reported that higher Numerical Rating Scale (NRS) scores are linked to greater fatty infiltration in specific deep extensor muscles, including the right semispinalis cervicis at C5‐6 and the right splenius capitis and right splenius cervicis and splenius capitis at C6‐7, whereas the left‐side muscles showed no such correlation.^37^ Moreover, individuals experiencing CINP tend to have higher levels of MFI in the multifidus, including the semispinalis cervicis.^39^ Regarding the curvature of the cervical spine, two studies found a negative correlation between cervical lordosis and the extent of fat infiltration in the cervical extensor muscles.^37^, ^38^ Furthermore, an investigation of the relationship between fat infiltration and the level of neck disability was shown,^36^, ^37^, ^38^ in which more significant fat infiltration in the cervical extensor muscles was related to poorer clinical neck disability index (NDI) and Northwick Park Questionnaire (NPQ) scores. However, there was a conflicting correlation between fatty infiltration and neck disability in the flexor muscles (longus colli and longus capitis). One study reported that the percentage of fatty infiltration at the right longus colli and longus capitis at C5‐6 and the left longus colli and longus capitis at C6‐7 had a significant positive correlation with the NPQ score,^37^ whereas another explained that there was no correlation between the NDI score and the percentage of infiltration of the longus colli muscle.^36^ In conclusion, the studies reviewed demonstrated a significant association between increased MFI in the cervical extensor and higher pain intensity, as well as poorer neck disability outcomes. However, findings on the relationship between MFI in the cervical flexors and neck disability levels remain inconsistent, warranting further investigation in future studies. Additionally, no correlation was observed between MFI in any cervical muscles and cervical lordosis.

## DISCUSSION

This review revealed evidence of a correlation between fat infiltration and neck pain characteristics, such as pain intensity and neck disability. However, no correlation was observed between MFI and cervical lordosis. This suggests that the presence of fat infiltration could potentially be used as a biomarker for chronic NSNP. It is important to note that not all the studies included in the analysis investigated the relationship between fatty infiltration and these specific aspects. Specifically, out of the four studies, one did not assess neck pain levels, another did not explore neck disability levels, and two did not measure neck alignment.

This review found evidence that MFI can accumulate in the flexor and extensor muscles of individuals with chronic NSNP. An increased MFI in the deep flexor and extensor muscles can directly affect the function and mechanics of the neck.^17^ This happens because deep muscles play a critical role in providing physical support to the spinal vertebral column and are essential for postural biomechanics, proprioception, and fine motor control.^40^ Moreover, fatty infiltration has also been observed in various spinal pathologies, including WAD.^26^, ^41^, ^42^, ^43^ Disuse results from psychological factors such as fear avoidance or passive pain‐coping styles that lead to accumulated fat infiltration in the neck muscles.^44^ However, this phenomenon has complex mechanisms that may relate to neuropsychological origins or skeletal muscle factors. Thus, further investigation is still needed to draw accurate conclusions.^45^, ^46^ Additionally, there is evidence that strength training for over 12 weeks can decrease MFI in the thigh muscles of adults with hip‐related groin pain.^47^ These findings suggest that future research should investigate interventions that might have the potential to reduce MFI in the flexor and extensor cervical muscles.

This study reported a correlation between fat infiltration in the cervical flexor and extensor muscles and higher pain levels in patients with NSNP and CINP, as measured using the visual analogue scale and NRS.^37^, ^38^, ^39^ Kim et al.^38^ explained that greater fat infiltration in the lower cervical extensor muscles was associated with decreased neck function and increased neck pain. This may be due to the axial load on the head and neck transmitted downward that may cause pain around the neck.^48^ Moreover, a study by Huang et al.^37^ revealed a significant correlation between fatty infiltration and NRS scores in patients with NSNP. Notably, this association was predominantly observed in the right semispinalis cervicis, semispinalis capitis, and splenius cervicis and splenius capitis muscles at the lower cervical segments, whereas no such correlation was noted in the corresponding muscles at the left side. These findings imply that MFI may occur asymmetrically and is not merely a consequence of general disuse. Instead, factors such as habitual posture, dominant‐side activity, or neuromuscular control may also play a role in this selective pattern.^37^ Additionally, Snodgrass et al.^39^ found that there was greater fat infiltration in the multifidus and semispinalis cervicis muscles in the pain group than in the asymptomatic group. This suggests a correlation between fatty infiltration in the deep cervical muscles and symptoms of neck pain. Physiotherapists can leverage this insight to prioritize examinations of the deep cervical muscles and administer targeted treatments to reduce fat accumulation. Ultimately, this focused approach could lead to improvements in both pain levels and neck disability for patients.

According to recent studies,^36^, ^37^, ^38^ there is a correlation between the disability level measured using the NDI and NPQ scores and the amount of fat infiltration in the extensor muscles, specifically the multifidus muscle, in people with NSNP. Similar findings have also been revealed by participants with WAD and posterior longitudinal ligament.^49^, ^50^ These findings may be explained by pathological conditions that include the natural degeneration of muscle, general disuse, response to direct damage, denervation, and demyelination.^38^ However, Elliot et al.^51^ explained that participants with chronic insidious‐onset neck pain had notably lower NDI scores compared to those with chronic WAD. They explained that if disuse was the primary or sole cause of fatty infiltrate, it would be expected to be present in those experiencing neck pain for a longer duration. Moreover, they suggested that the factors related to disuse are more closely linked to higher levels of pain and disability, which affect activity levels, rather than the longer duration of symptoms. In conclusion, they proposed that fatty infiltrates in the cervical extensor musculature were not a characteristic feature of insidious onset neck pain but were present only in patients with WAD.

Furthermore, there was a conflicting correlation between fatty infiltration and the level of neck disability in the flexor muscles (longus colli and longus capitis). Huang et al. (2022) reported that the percentage of fatty infiltration in the cervical flexor muscles was significantly positively correlated with the NPQ score.^37^ A study by Paliwal et al.^17^ supported this finding, showing that patients with degenerative cervical myelopathy with elevated Nuric scores had significantly higher MFI in the cervical flexors than healthy controls. Patients with this condition often have cervical misalignment, leading to forward head posturing, which can result in shortened cervical flexors and an increased MFI.^52^, ^53^, ^54^, ^55^ In contrast, Grondin et al.^36^ reported no correlation between NDI scores and the percentage of fat accumulation in the longus colli muscle. They suggested that this may be because deep flexor muscles are less prone to fat accumulation compared to deep extensor muscles.

The association between fat infiltration and cervical curvature has been investigated in two studies ).^37^, ^38^ These studies observed a negative correlation between fat infiltration of the cervical extensor muscles and cervical lordosis. One study suggested that this correlation might be due to relatively uniform patient groups and small sample sizes, highlighting the need for larger and more diverse patient populations.^37^ However, one study did not provide a reason for the observed correlation.^38^ In contrast, Liu et al.^56^ found a potential correlation between disc bulge at the stenosis segment and reduction in lumbar lordosis with fatty infiltration in the multifidus muscles in degenerative lumbar spinal stenosis. They suggested that changes in sagittal alignment could increase strain on the affected multifidus muscle, leading to exacerbation of MFI.

It is important to note that most of the studies included in the review exhibit a low risk of bias, which enhances the reliability of our findings. Additionally, to minimize measurement bias, all study participants underwent standardized assessment using validated instruments, such as MRI, to ensure consistency and accuracy in measuring MFI.

Nonetheless, this review has several limitations. The lack of homogeneity among the included studies, particularly in participant characteristics (age, body mass index, and gender), and muscle groups, may have influenced the results, as these factors can act as potential confounders. Additionally, data extraction was performed by only one reviewer, which may introduce bias and reduce reproducibility. Furthermore, although most studies exhibited a low risk of bias, they were all cross‐sectional in design, limiting the ability to establish causality between MFI and clinical outcomes. Moreover, there are several reasons for the absence of a meta‐analysis. First, only four studies met the inclusion criteria, which is insufficient to conduct a robust meta‐analysis. Second, there was considerable variation in the outcome measurements across these studies. Furthermore, the findings from these studies were inconsistent; two of them reported conflicting results regarding the relationship between MFI and clinical outcomes, which impeded the ability to derive a combined estimate. Finally, the search strategy was developed without formal consultation with a medical librarian, which may have affected the comprehensiveness of the search terms employed.

The findings of this review highlight the necessity for further research into the relationship between MFI and pain, disability, and cervical alignment. It is advisable to investigate longitudinal or experimental study designs to establish causal relationships and deepen our understanding of MFI‐related dysfunctions. These findings underscore the importance of considering rehabilitation programs targeting MFI reduction and improved muscle function, which could potentially alleviate symptoms and enhance cervical alignment.

## CONCLUSION

This systematic review found a correlation between increased MFI in cervical extensor muscles and higher pain intensity and greater disability in individuals with NSNP. However, the relationship between MFI in the cervical flexors and neck disability levels remains inconsistent. Additionally, no correlation was observed between MFI in any cervical muscles and cervical lordosis. These results highlight the need for further investigation to better understand these associations and causality, as well as to explore interventions aimed at reducing MFI in neck muscles and their potential clinical benefits.

## DISCLOSURE

No potential conflict of interest was reported by the authors.

## Supporting information


**Appendix S1.** Supporting Information.

## Data Availability

The authors confirm that all data supporting the findings of this study are available within the article and its supplementary materials.

## References

[1] Safiri S , Kolahi AA , Hoy D , et al. Global, regional, and national burden of neck pain in the general population, 1990‐2017: systematic analysis of the Global Burden of Disease study 2017. BMJ (Clinical Research Ed). 2020;368:m791. doi:10.1136/bmj.m791 PMC7249252 32217608

[2] Kazeminasab S , Nejadghaderi SA , Amiri P , et al. Neck pain: global epidemiology, trends and risk factors. BMC Musculoskelet Disord. 2022;23(1):26. doi:10.1186/s12891-021-04957-4 34980079 PMC8725362

[3] Mesas AE , González AD , Mesas CE , de Andrade SM , Magro IS , del Llano J . The association of chronic neck pain, low back pain, and migraine with absenteeism due to health problems in Spanish workers. Spine. 2014;39(15):1243‐1253. doi:10.1097/BRS.0000000000000387 24825151

[4] Côté P , Cassidy DJ , Carroll LJ , Kristman V . The annual incidence and course of neck pain in the general population: a population‐based cohort study. Pain. 2004;112(3):267‐273. doi:10.1016/j.pain.2004.09.004 15561381

[5] Minghelli B . Musculoskeletal spine pain in adolescents: epidemiology of non‐specific neck and low back pain and risk factors. J Orthop Sci. 2020;25(5):776‐780. doi:10.1016/j.jos.2019.10.008 31708228

[6] Jun D , Zoe M , Johnston V , O'Leary S . Physical risk factors for developing non‐specific neck pain in office workers: a systematic review and meta‐analysis. Int Arch Occup Environ Health. 2017;90(5):373‐410. doi:10.1007/s00420-017-1205-3 28224291

[7] Correa‐de‐Araujo R , Addison O , Miljkovic I , et al. Myosteatosis in the context of skeletal muscle function deficit: an interdisciplinary workshop at the National Institute on Aging. Front Physiol. 2020;11:963. doi:10.3389/fphys.2020.00963 32903666 PMC7438777

[8] Pagano AF , Demangel R , Brioche T , et al. Muscle regeneration with intermuscular adipose tissue (IMAT) accumulation is modulated by mechanical constraints. PLoS One. 2015;10(12):e0144230. doi:10.1371/journal.pone.0144230 26629696 PMC4668059

[9] Zuo YQ , Gao ZH , Wang Z , et al. Utility of multidetector computed tomography quantitative measurements in identifying sarcopenia: a propensity score matched study. Skeletal Radiol. 2022;51(6):1303‐1312. doi:10.1007/s00256-021-03953-y 34757481

[10] Addison O , Marcus RL , Lastayo PC , Ryan AS . Intermuscular fat: a review of the consequences and causes. Int J Endocrinol. 2014;2014:309570. doi:10.1155/2014/309570 24527032 PMC3910392

[11] Beasley LE , Koster A , Newman AB , et al. Inflammation and race and gender differences in computerized tomography‐measured adipose depots. Obesity (Silver Spring, Md). 2009;17(5):1062‐1069. doi:10.1038/oby.2008.627 19165157 PMC3268118

[12] Hausman GJ , Basu U , Du M , Fernyhough‐Culver M , Dodson MV . Intermuscular and intramuscular adipose tissues: bad vs. good adipose tissues. Adipocyte. 2014;3(4):242‐255. doi:10.4161/adip.28546 26317048 PMC4550684

[13] Kalichman L , Carmeli E , Been E . The association between imaging parameters of the paraspinal muscles, spinal degeneration, and low back pain. Biomed Res Int. 2017;2017:2562957. doi:10.1155/2017/2562957 28409152 PMC5376928

[14] Ekşi MŞ , Özcan‐Ekşi EE . Fatty infiltration of the erector spinae at the upper lumbar spine could be a landmark for low back pain. Pain Pract. 2024;24(2):278‐287. doi:10.1111/papr.13302 37830410

[15] Ekşi MŞ , Öztaş UO , Topaloğlu F , et al. Erector spinae could be the game changer in surgical decision‐making in patients with lumbar spondylolisthesis: a cross‐sectional analysis of an age‐, sex‐, subtype‐, level‐matched patients with similar spinopelvic parameters received surgical or conservative management. Eur Spine J. 2024;33(10):3715‐3723. doi:10.1007/s00586-024-08341-3 38809440

[16] Özcan‐Ekşi EE , Ekşi M , Turgut VU , Canbolat Ç , Pamir MN . Reciprocal relationship between multifidus and psoas at L4‐L5 level in women with low back pain. Br J Neurosurg. 2020;35:220‐228. doi:10.1080/02688697.2020.1783434 32576034

[17] Paliwal M , Weber KA 2nd , Smith AC , et al. Fatty infiltration in cervical flexors and extensors in patients with degenerative cervical myelopathy using a multi‐muscle segmentation model. PLoS One. 2021;16(6):e0253863. doi:10.1371/journal.pone.0253863 34170961 PMC8232539

[18] Cloney M , Smith AC , Coffey T , et al. Fatty infiltration of the cervical multifidus musculature and their clinical correlates in spondylotic myelopathy. J Clin Neurosci. 2018;57:208‐213. doi:10.1016/j.jocn.2018.03.028 30243599

[19] Elysee JC , Lovecchio F , Lafage R , et al. The relationship of global sagittal malalignment to fatty infiltration in the aging spine. Eur Spine J. 2021;30(9):2480‐2485. doi:10.1007/s00586-021-06759-7 33609190

[20] Chianca V , Vincenzo B , Cuocolo R , et al. MRI quantitative evaluation of muscle fatty infiltration. Magnetochemistry. 2023;9(4):111. doi:10.3390/magnetochemistry9040111

[21] Battaglia PJ , Maeda Y , Welk A , Hough B , Kettner N . Reliability of the Goutallier classification in quantifying muscle fatty degeneration in the lumbar multifidus using magnetic resonance imaging. J Manip Physiol Ther. 2014;37(3):190‐197. doi:10.1016/j.jmpt.2013.12.010 24630770

[22] Upadhyay B , Toms AP . CT and MRI evaluation of paraspinal muscle degeneration. European Congress of Radiology. European Society of Radiology; 2015:1‐15.

[23] Lee JC , Cha JG , Kim Y , Kim YI , Shin BJ . Quantitative analysis of back muscle degeneration in the patients with the degenerative lumbar flat back using a digital image analysis: comparison with the normal controls. Spine. 2008;33(3):318‐325. doi:10.1097/BRS.0b013e318162458f 18303466

[24] Keller A , Gunderson R , Reikerås O , Brox JI . Reliability of computed tomography measurements of paraspinal muscle cross‐sectional area and density in patients with chronic low back pain. Spine. 2003;28(13):1455‐1460. doi:10.1097/01.BRS.0000067094.55003.AD 12838105

[25] Prasetyo M , Nindita N , Murdana IN , Prihartono J , Setiawan SI . Computed tomography evaluation of fat infiltration ratio of the multifidus muscle in chronic low back pain patients. Eur J Radiol Open. 2020;7:100293. doi:10.1016/j.ejro.2020.100293 33304941 PMC7718153

[26] Park BK , Hong SH , Jeong WK . Effectiveness of ultrasound in evaluation of fatty infiltration in rotator cuff muscles. Clin Orthop Surg. 2020;12(1):76‐85. doi:10.4055/cios.2020.12.1.76 32117542 PMC7031432

[27] Pedroso MG , de Almeida AC , Aily JB , de Noronha M , Mattiello SM . Fatty infiltration in the thigh muscles in knee osteoarthritis: a systematic review and meta‐analysis. Rheumatol Int. 2019;39(4):627‐635. doi:10.1007/s00296-019-04271-2 30852623

[28] De Pauw R , Coppieters I , Kregel J , De Meulemeester K , Danneels L , Cagnie B . Does muscle morphology change in chronic neck pain patients? ‐ a systematic review. Man Ther. 2016;22:42‐49. doi:10.1016/j.math.2015.11.006 26724855

[29] Peng Q , Zhang Y , Yang S , et al. Morphologic changes of cervical musculature in relation to chronic nonspecific neck pain: a systematic review and meta‐analysis. World Neurosurg. 2022;168:79‐88. doi:10.1016/j.wneu.2022.09.057 36126892

[30] Ebenbichler GR , Oddsson LIE , Kollmitzer J , Erim Z . Sensory‐motor control of the lower back: implications for rehabilitation. Med Sci Sports Exerc. 2001;33(11):1889‐1898. doi:10.1097/00005768-200111000-00014 11689740

[31] Freeman MD , Woodham MA , Woodham AW . The role of the lumbar multifidus in chronic low back pain: a review. PM R. 2010;2(2):142‐167. doi:10.1016/j.pmrj.2009.11.006 20193941

[32] Hodges PW , Tucker K . Moving differently in pain: a new theory to explain the adaptation to pain. Pain. 2011;152(3 Suppl):S90‐S98. doi:10.1016/j.pain.2010.10.020 21087823

[33] Moher D , Liberati A , Tetzlaff J , Altman DG , PRISMA Group . Preferred reporting items for systematic reviews and meta‐analyses: the PRISMA statement. PLoS Med. 2009;6(7):e1000097. doi:10.1371/journal.pmed.1000097 19621072 PMC2707599

[34] Moola S , Munn Z , Tufanaru C , et al. Systematic reviews of etiology and risk. In: Aromataris E , Lockwood C , Porritt K , Pilla B , Jordan Z , eds. JBI Manual for Evidence Synthesis. JBI; 2020.

[35] Franco A , Vidigal MTC , Oliveira MND , Nascimento CTDJS , Silva RFD , Paranhos LR . Evidence‐based mapping of third molar techniques for age estimation applied to Brazilian adolescents – a systematic review. Res Soc Dev. 2020;9:e9339109395. doi:10.33448/rsd-v9i10.9395

[36] Grondin F , Freppel S , Jull G , Gérard T , Caderby T , Peyrot N . Fat infiltration of multifidus muscle is correlated with neck disability in patients with non‐specific chronic neck pain. J Clin Med. 2022;11(19):5522. doi:10.3390/jcm11195522 36233390 PMC9571215

[37] Huang Z , Bai Z , Yan J , et al. Association between muscle morphology changes, cervical spine degeneration, and clinical features in patients with chronic nonspecific neck pain: a magnetic resonance imaging analysis. World Neurosurg. 2022;159:e273‐e284. doi:10.1016/j.wneu.2021.12.041 34929370

[38] Kim CY , Lee SM , Lim SA , Choi YS . Impact of fat infiltration in cervical extensor muscles on cervical lordosis and neck pain: a Cross‐sectional study. Clin Orthop Surg. 2018;10(2):197‐203. doi:10.4055/cios.2018.10.2.197 29854343 PMC5964268

[39] Snodgrass SJ , Stanwell P , Weber KA , et al. Greater muscle volume and muscle fat infiltrate in the deep cervical spine extensor muscles (multifidus with semispinalis cervicis) in individuals with chronic idiopathic neck pain compared to age and sex‐matched asymptomatic controls: a cross‐sectional study. BMC Musculoskelet Disord. 2022;23(1):973. doi:10.1186/s12891-022-05924-3 36357864 PMC9647973

[40] Boyd‐Clark LC , Briggs CA , Galea MP . Muscle spindle distribution, morphology, and density in longus colli and multifidus muscles of the cervical spine. Spine. 2002;27(7):694‐701. doi:10.1097/00007632-200204010-00005 11923661

[41] Elliott J , Jull G , Noteboom JT , Darnell R , Galloway G , Gibbon WW . Fatty infiltration in the cervical extensor muscles in persistent whiplash‐associated disorders: a magnetic resonance imaging analysis. Spine. 2006;31(22):E847‐E855. doi:10.1097/01.brs.0000240841.07050.34 17047533

[42] Smith AC , Albin SR , Abbott R , et al. Confirming the geography of fatty infiltration in the deep cervical extensor muscles in whiplash recovery. Sci Rep. 2020;10(1):11471. doi:10.1038/s41598-020-68452-x 32651447 PMC7351986

[43] Smith AC , Parrish TB , Hoggarth MA , et al. Potential associations between chronic whiplash and incomplete spinal cord injury. Spinal Cord Ser Cases. 2015;1:15024. doi:10.1038/scsandc.2015.24 PMC5019487 27630770

[44] Vlaeyen JWS , Linton SJ . Fear‐avoidance model of chronic musculoskeletal pain: 12 years on. Pain. 2012;153(6):1144‐1147. doi:10.1016/j.pain.2011.12.009 22321917

[45] Clark BC . In vivo alterations in skeletal muscle form and function after disuse atrophy. Med Sci Sports Exerc. 2009;41(10):1869‐1875. doi:10.1249/MSS.0b013e3181a645a6 19727027

[46] Elliott JM , Courtney DM , Rademaker A , Pinto D , Sterling MM , Parrish TB . The rapid and progressive degeneration of the cervical multifidus in whiplash: an MRI study of fatty infiltration. Spine. 2015;40(12):E694‐E700. doi:10.1097/BRS.0000000000000891 25785961 PMC4466088

[47] Koch K , Semciw AI , Commean PK , et al. Comparison between movement pattern training and strengthening on muscle volume, muscle fat, and strength in patients with hip‐related groin pain: an exploratory analysis. J Orthop Res. 2022;40(6):1375‐1386. doi:10.1002/jor.25158 34370330 PMC8825882

[48] Harrison DE , Harrison DD , Janik TJ , William Jones E , Cailliet R , Normand M . Comparison of axial and flexural stresses in lordosis and three buckled configurations of the cervical spine. Clin Biomech (Bristol, Avon). 2001;16(4):276‐284. doi:10.1016/s0268-0033(01)00006-7 11358614

[49] Abbott R , Pedler A , Sterling M , et al. The geography of fatty infiltrates within the cervical multifidus and semispinalis cervicis in individuals with chronic whiplash‐associated disorders. J Orthop Sports Phys Ther. 2015;45(4):281‐288. doi:10.2519/jospt.2015.5719 25739843 PMC7223031

[50] Doi T , Ohtomo N , Oguchi F , et al. Association between deep posterior cervical paraspinal muscle morphology and clinical features in patients with cervical ossification of the posterior longitudinal ligament. Global Spine Journal. 2023;13(1):8‐16. doi:10.1177/2192568221989655 33504203 PMC9837499

[51] Elliott J , Sterling M , Noteboom JT , Darnell R , Galloway G , Jull G . Fatty infiltrate in the cervical extensor muscles is not a feature of chronic, insidious‐onset neck pain. Clin Radiol. 2008;63(6):681‐687. doi:10.1016/j.crad.2007.11.011 18455560

[52] Ames CP , Blondel B , Scheer JK , et al. Cervical radiographical alignment: comprehensive assessment techniques and potential importance in cervical myelopathy. Spine. 2013;38(22 Suppl 1):S149‐S160. doi:10.1097/BRS.0b013e3182a7f449 24113358

[53] Bodine SC . Disuse‐induced muscle wasting. Int J Biochem Cell Biol. 2013;45(10):2200‐2208. doi:10.1016/j.biocel.2013.06.011 23800384 PMC3856924

[54] Elliott JM , O'Leary S , Sterling M , Hendrikz J , Pedler A , Jull G . Magnetic resonance imaging findings of fatty infiltrate in the cervical flexors in chronic whiplash. Spine. 2010;35(9):948‐954. doi:10.1097/BRS.0b013e3181bb0e55 20118837

[55] Mohanty C , Massicotte EM , Fehlings MG , Shamji MF . Association of preoperative cervical spine alignment with spinal cord magnetic resonance imaging hyperintensity and myelopathy severity: analysis of a series of 124 cases. Spine. 2015;40(1):11‐16. doi:10.1097/BRS.0000000000000670 25341991

[56] Liu Y , Liu Y , Hai Y , Li G , Liu T , Wang Y . Lumbar lordosis reduction and disc bulge may correlate with multifidus muscle fatty infiltration in patients with single‐segment degenerative lumbar spinal stenosis. Clin Neurol Neurosurg. 2020;189:105629. doi:10.1016/j.clineuro.2019.105629 31830678

