# Supplementary material for: Muscle fat infiltration and its relation with pain intensity, disability, and cervical curvature in individuals with nonspecific neck pain: A systematic review study
Source: PM R. 2026 Jan 12;18(7):793–802. doi: 10.1002/pmrj.70064 (PMC13358439; doi:10.1002/pmrj.70064)
Supplement: Supplementary file 1 — Appendix S1. Supporting Information. [file PMRJ-18-793-s001.docx]

**Appendix**

Search strategy for each database:

**Pubmed**

**(((((neck pain[MeSH Terms]) OR (chronic neck pain)) OR (non-specific neck pain OR NSNP)) OR (chronic non-specific neck pain OR CNSNP)) AND (medical imaging OR magnetic resonance imaging OR MRI OR computer tomography OR CT OR ultrasonography)) AND (muscle fat infiltration OR "fat infiltration" OR FI OR "fat infiltration area" OR FIA OR neck muscl*)**

**CINAHL**

neck pain OR chronic neck pain OR non-specific neck pain OR NSNP OR chronic non-specific neck pain OR CNSNP

AND

medical imaging OR magnetic resonance imaging OR MRI OR computer tomography OR CT OR ultrasonography

AND

muscle fat* infiltration OR "fat* infiltration" OR FI OR "fat* infiltration area" OR FIA OR neck muscl*

**EBSCO host**

neck pain OR chronic neck pain OR non-specific neck pain OR NSNP OR chronic non-specific neck pain OR CNSNP

AND

medical imaging OR magnetic resonance imaging OR MRI OR computer tomography OR CT OR ultrasonography

AND

muscle fat* infiltration OR "fat* infiltration" OR FI OR "fat* infiltration area" OR FIA OR neck muscl*

**Web of Science**

neck pain OR chronic neck pain OR non-specific neck pain OR nsip OR chronic non-specific neck pain OR cnsns (All Fields) and medical imaging OR magnetic resonance imaging OR MRI OR computer tomography OR CT OR ultrasonography (All Fields) and muscle fat* infiltration OR "fat* infiltration" OR FI OR "fat* infiltration area" OR FIA OR neck muscl* (All Fields)
